# Male reproductive health as a sentinel for environmental endocrine disruption

**DOI:** 10.1186/1751-0147-54-S1-S1

**Published:** 2012-02-24

**Authors:** Jorma Toppari

**Affiliations:** 1 Departments of Paediatrics and Physiology, University of Turku, FI-20520 Turku, Finland

## 

Effects of endocrine disruptors on experimental animals and wildlife are well established. Antiandrogens and estrogens cause reproductive disorders, such as cryptorchidism, hypospadias, testicular dysgenesis and subfertility. This raises a natural question whether these disorders in humans are also caused by endocrine disrupters and whether they should alert us of harmful exposures. While we still do not know the answer to this question, we know that the incidence of testicular cancer has rapidly increased over two generations, and the birth rates of hypospadias and cryptorchidism are alarmingly high. Furthermore, semen quality of young European men remains very poor at the moment. We have analyzed the association of cryptorchidism with exposure to several endocrine disruptors. This kind of studies cannot prove any causality. However, we have found a weak positive association of cryptorchidism with exposure to chlorinated pesticides, dioxins and furans, and polybrominated diphenyl ethers. It has become obvious that there is no individual compound that could be linked to etiology of cryptorchidism, but rather a mixture of several chemicals can cause the effect in genetically susceptible individuals. Modern systems biological approaches are needed to deal with complex exposure scenarios and genetic variability.

Supported by the Academy of Finland, EU Fp7 Environment DEER, Sigrid Juselius Foundation, and Turku University Hospital.

